# Oral Treatment With Heat Shock Protein 65‐Producing *Lactococcus lactis* Induces Regulatory T Cells, Modulating Inflammatory Response in *Leishmania braziliensis* Infection

**DOI:** 10.1111/imm.70022

**Published:** 2025-07-31

**Authors:** Camila Mattos Andrade, Ítalo da Silva Gonçalves, Maria Luiza das Neves Nascimento, Washington Luís Conrado Santos, Vasco Ariston Azevedo, Deborah Bittencourt Mothé, Juliana Perrone Menezes Fullam, Patrícia Sampaio Tavares Veras, Natalia Machado Tavares, Tatiani Uceli Maioli, Ana Maria Caetano Faria, Cláudia Ida Brodskyn

**Affiliations:** ^1^ Laboratory of Host‐Pathogen Interaction and Epidemiology (LAIPHE) Gonçalo Moniz Institute, Oswaldo Cruz Foundation Salvador Bahia Brazil; ^2^ Laboratory of Molecular and Structural Pathology (LAPEM) Gonçalo Moniz Institute, Oswaldo Cruz Foundation Salvador Bahia Brazil; ^3^ Deparment of Pathology and Legal Medicine School of Medicine of Federal University of Bahia Salvador Bahia Brazil; ^4^ Deparment of Genetics, Ecology and Evolution Biological Sciences Institute, Federal University of Minas Gerais Belo Horizonte Minas Gerais Brazil; ^5^ National Institute of Science and Technology of Tropical Diseases (INCT‐DT), National Council for Scientific Research and Development (CNPq) Salvador Bahia Brazil; ^6^ Institute of Investigation in Immunology (iii), National Institute of Science and Technology (INCT) São Paulo São Paulo Brazil; ^7^ Department of Nutrition Nursing School, Federal University of Minas Gerais Belo Horizonte Minas Gerais Brazil; ^8^ Department of Biochemistry and Immunology Biological Sciences Institute, Federal University of Minas Gerais Belo Horizonte Minas Gerais Brazil

**Keywords:** cutaneous leishmaniasis, HSP65, *Lactococcus lactis*, oral tolerance

## Abstract

Cutaneous leishmaniasis (CL), a neglected tropical disease prevalent in Brazil, is caused by *Leishmania braziliensis* ( *L. braziliensis* ) and is marked by ulcerative skin lesions and an exacerbated Th1‐driven inflammatory response. This study investigates the therapeutic potential of oral tolerance (OT) induced by a genetically modified strain of *Lactococcus lactis* ( *L. lactis* ) producing heat shock protein 65 (HSP65) from *Mycobacterium leprae* in a murine model of CL. BALB/c mice were infected with *L. braziliensis* and treated orally with HSP65‐producing *L. lactis* or control *L. lactis* (empty vector) for four consecutive days, starting at 4 weeks post‐infection. Mice receiving HSP65‐producing *L. lactis* showed reduced lesion size and parasite burden. Cytokine analysis in draining lymph nodes revealed a shift from a pro‐inflammatory IFN‐γ response to an increased IL‐10 production, correlating with milder inflammation and less tissue damage. Additionally, the treatment promoted an increase in regulatory T cells (Tregs), including CD4^+^CD25^+^FOXP3^+^ and CD4^+^LAP^+^ (membrane‐associated TGF‐β) cells in the draining lymph nodes. This therapeutic effect was not observed in a more severe model of CL using Leishmania major. This study underscores the potential of oral tolerance induction using HSP65‐producing *L. lactis* as a promising immunoregulatory therapeutic approach for some chronic inflammatory infections, mainly those that display a primed balance in immune response.

## Graphical abstract

Oral treatment with HSP65‐producing *Lactococcus lactis* after *Leishmania braziliensis* infection modulates inflammation by promoting lesion resolution through increased IL‐10 production and expansion of regulatory T cells. These findings highlight the potential of oral tolerance induction as a promising therapeutic strategy for chronic inflammatory infections, particularly those with an immune response displaying a primed balance.

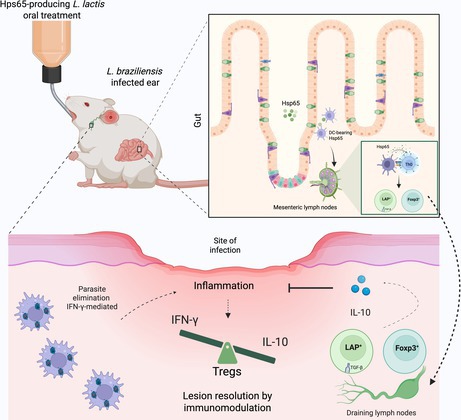

## 1 Introduction

Cutaneous leishmaniasis (CL) induced by *Leishmania braziliensis* ( *L. braziliensis* ) presents a complex clinical profile characterised by ulcerated chronic lesions [1]. The human immune response in *L. braziliensis* ‐induced cutaneous leishmaniasis is characterised by an exacerbated Th1 cellular immune response and involves the high production of pro‐inflammatory cytokines, contributing to tissue destruction despite the low presence of parasites [1, 2, 3]. Therefore, it is crucial to explore therapeutic alternatives that modulate inflammation to control disease exacerbation while maintaining a sufficient inflammatory response for parasite elimination. On the other hand, a severe model of disease is observed in BALB/c mice infected with *Leishmania major* ( *L. major* ), with a predominance of Th2 response, characterised by a high production of IL‐4 and IL‐10, and low production of IFN‐γ, demonstrating an alternative inflammatory response with a high number of parasites [4].

Oral tolerance (OT), a naturally occurring immunological phenomenon that suppresses the inflammatory immune response to exogenous proteins that are administered by oral route, emerges as a promising therapeutic approach [5]. OT induction effectively generates regulatory T cells (Tregs), pivotal in inhibiting inflammatory immune responses [6, 7]. The continuous ingestion of antigens, exemplified by the myelin oligodendrocyte glycoprotein (MOG) [8] and insulin [9], has been shown to induce antigen‐specific Treg responses. It is noteworthy that OT can suppress bystander antigens, particularly in cases where the target antigen is unknown, or multiple antigens contribute to pathology [10].

It has been demonstrated that Heat Shock Proteins (HSPs) present similarities across different species, and some of them, such as mammalian HSP60, cause down regulation in the inflammatory responses in vitro by two different mechanisms: as a ligand for TLR2, which inhibits the migration of effector T cells to different inflammatory sites [11] and as a self‐antigen recognised by regulatory T cells, inducing anti‐inflammatory effects [12]. In our study, we employed HSP65 from *Mycobacterium leprae* ( *M. leprae* ), similar to mammalian HSP60, that has been used to induce oral tolerance [13, 14, 15, 16, 17]. We employ the non‐pathogenic, colonising bacteria *Lactococcus lactis* ( *L. lactis* ) to optimise protein delivery, capitalising on its probiotic properties [18, 19]. Recombinant *L. lactis* has successfully induced cytokine secretion in experimental colitis models, presenting a cost‐effective method for controlled protein release [20, 21]. Numerous studies have demonstrated that oral administration of recombinant *L. lactis* producing HSP65 derived from *M. leprae* promotes the prevention of autoimmune and inflammatory diseases through OT induction [22, 23, 24, 25].

Our previous research showed for the first time that OT induced by prior administration of HSP65‐producing *L. lactis* in the CL mouse model was able to mitigate inflammation caused by a pathogen [26]. Consequently, this study aims to advance the therapeutic potential by directly delivering *M. leprae* ‐derived HSP65 into the intestinal mucosa via a recombinant *L. lactis* strain after lesion development induced by *L. braziliensis* and *L. major* infection. Our goal is to develop an innovative immunomodulatory strategy capable of modulating the inflammatory effects associated with *L. braziliensis* and *L. major* infections, thereby contributing to the search for therapeutic alternatives in the pursuit of effective CL management.

## 2 Results

### 2.1 Oral Administration of HSP65‐Producing *L. lactis* Reduces Lesion Size and Inflammation in Mice Infected With *L. braziliensis*

**FIGURE 1:**
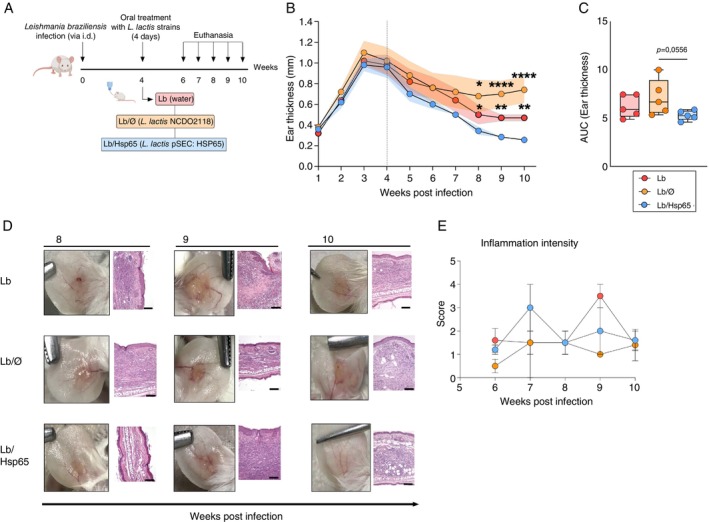
Lesion progression in *L. braziliensis* ‐infected BALB/c mice treated with HSP65‐producing *L. lactis* . BALB/c mice were infected in the ear with *L. braziliensis* metacyclic promastigotes then, 4 weeks post‐infection, fed with water (Lb), GM17 medium containing empty vector‐bearing *L. lactis* (Lb/Ø) or XM17 medium containing HSP65‐producing *L. lactis* (Lb/HSP65) for four consecutive days. Ear thickness was measured weekly with a digital calliper. (A) Experimental design in BALB/c mice. (B) Ear thickness kinetics and we calculated the area under curve (C) with ear thickness data from each group. (D) Representative lesion histopathological aspects from 8 to 10 weeks after infection in respective groups. Ears were collected, fixed in 10% formaldehyde, processed, then stained with haematoxylin and eosin. (E) Inflammation intensity score kinetics. Histopathological changes were evaluated, including inflammation intensity. We determined the score according to intensity: 0 (absence), 1 (presence of 1%–25%), 2 (presence of 25%–50%) and 3 (> 50%). We used a healthy animal ear as a negative control. Data are representative of means from each group. (B–E) Data represented by median and interquartile range from each group of three independent experiments involving five animals per group. The Kruskal–Wallis test with Dunn's post‐test was used. *p < 0.05; **p < 0.001.

To evaluate the immunomodulatory potential of oral treatment with HSP65‐producing *L. lactis* on lesion development caused by *L. braziliensis* , BALB/c mice were infected in the ear with *L. braziliensis* metacyclic promastigotes. Four weeks post‐infection, mice were fed with water (Lb), GM17 medium containing empty vector‐bearing *L. lactis* (Lb/Ø) or XM17 medium containing HSP65‐producing *L. lactis* (Lb/HSP65) for four consecutive days, from day 28 to 31 post infection (Figure 1A). Mice from the Lb/HSP65 group developed smaller lesions at 5‐, 8‐, 9‐, and 10‐weeks post‐infection compared to the Lb and Lb/Ø groups (Figure 1B). Likewise, a significant reduction was observed in the area under the curve (AUC) values of ear thickness in mice from the Lb/HSP65 group compared to the control groups (Figure 1C).

Mice from the Lb/HSP65 group showed a less intense inflammatory infiltrate, with most animals showing the absence of ulcer formation compared to the other groups after 6 to 10 weeks of infection (Figures 1D and S1). In contrast, mice from the Lb and Lb/Ø groups exhibited an intense response with ulcerated lesions with raised edges from 6 to 8 weeks post‐infection (Figures 1D and S1). Regarding histopathological score analysis, although significant differences among the experimental groups were not observed, mice from the Lb/HSP65 group seemed to slightly early increase the inflammation intensity in ear lesions caused by *L. braziliensis* at 7 weeks of infection, followed by a decline in 8 to 10 weeks post‐infection, whereas the Lb group displayed a later inflammatory intensity response, characterising a chronic infection model (Figure 1E). Other histopathological parameters were evaluated, but significant differences were not observed among all experimental groups (Figure S2).

Our data suggest that oral administration of HSP65‐producing *L. lactis* after infection with *L. braziliensis* ameliorates disease chronicity, which we have associated with a less intense inflammatory profile observed in the lesions from mice treated with HSP65 in the final stage of infection.

### 2.2 Oral Administration of HSP65‐Producing *L. lactis* Effectively Controls Parasite Load in Mice Infected With *L. braziliensis*

**FIGURE 2:**
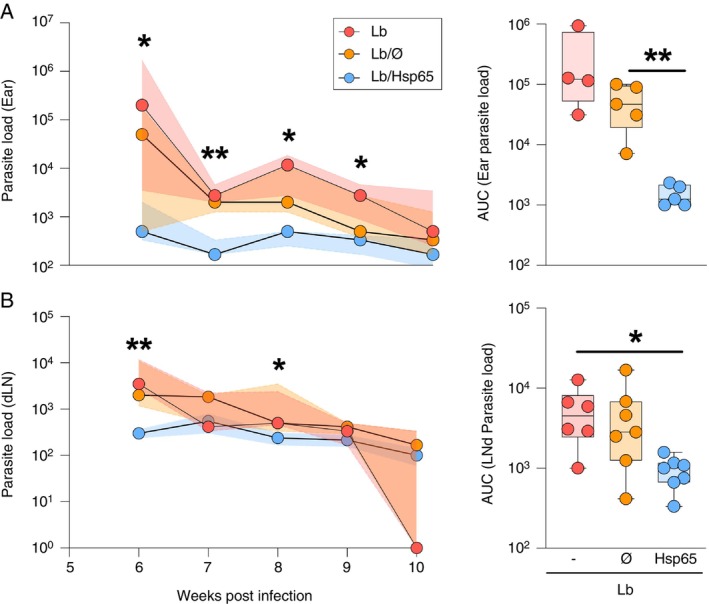
Parasite load kinetics in *L. braziliensis* ‐infected BALB/c mice treated with HSP65‐producing *L. lactis*. BALB/c mice were infected in the ear with *L. braziliensis* metacyclic promastigotes then, 4 weeks post‐infection, fed with water (Lb), GM17 medium containing empty vector‐bearing *L. lactis* (Lb/Ø) or XM17 medium containing HSP65‐producing *L. lactis* (Lb/HSP65) for four consecutive days. At 6–10 weeks post‐infection, ears and draining lymph nodes were collected to quantify the parasite load by limiting dilution. (A) Parasite load kinetics of the ear and (B) draining lymph nodes from Lb, Lb/Ø and Lb/HSP65 groups. We also calculated the area under curve using parasite load data of the ear (A‐right panel) and of the draining lymph nodes (B‐right panel) from each group. (A, B) Data are represented by the median and interquartile range for each group. Kruskal–Wallis with Dunn's post‐test was used. *p < 0.05 (between Lb and Lb/HSP65 groups at 6, 8 and 9 weeks in (A) and at 8 weeks in (B)) and **p < 0.001 [between Lb and Lb/HSP65 groups at 6 weeks (B) and 7 weeks in (A)].

We also investigated whether oral treatment with HSP65‐producing *L. lactis* post‐infection interfered with the number of parasites present in the lesions and draining lymph nodes. Our data demonstrated that oral treatment with HSP65 reduced the number of parasites in the ear at 6 to 9 weeks after infection compared to mice from the Lb group (Figure 2A). The Lb/HSP65 group showed significantly lower AUC values than Lb/Ø (Figure 2A, right panel). Parasite load reduction in draining lymph nodes was observed in animals from Lb/HSP65 at the 6‐ and 8‐weeks post‐infection compared to the other groups (Figure 2B). Correspondingly, Lb/HSP65 showed a smaller AUC than Lb (Figure 2B, right panel).

On the other hand, mice from Lb and Lb/Ø groups presented a higher number of parasites in the ear at 6 weeks post‐infection (Figure 2A). At 10 weeks post‐infection, all experimental groups presented few parasites at the sites of infection (ears and draining lymph nodes) (Figure 2A,B) but mice from Lb and Lb/Ø groups exhibited larger lesions than the Lb/HSP65 group (Figure 1B). This shows that ear thickness is also associated with the inflammatory response against infection rather than the presence of the parasite. Therefore, our data demonstrated that oral treatment with HSP65‐producing *L. lactis* did not interfere with the ability to eliminate the parasite, resulting in a reduction and control of the parasite proliferation at the sites of infection.

### 2.3 Oral Administration of HSP65‐Producing *L. lactis* Modulates Cytokine Production and Mitigates Tissue Damage in Mice Infected With *L. braziliensis*

**FIGURE 3:**
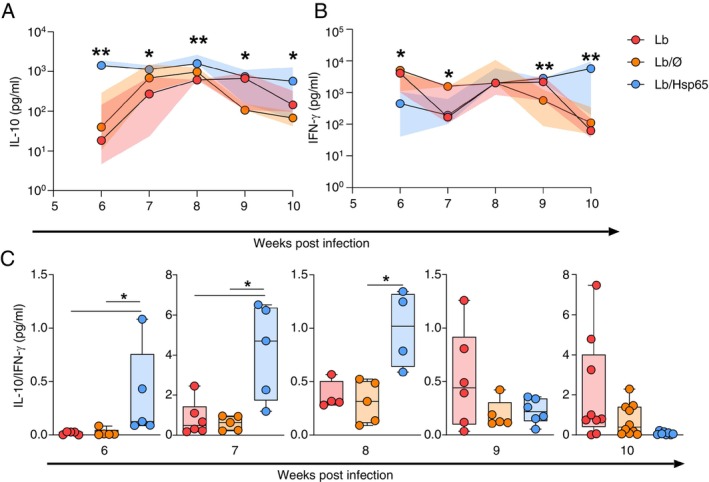
Pro‐ and anti‐inflammatory cytokine production kinetics and the ratio of IL/‐10/IFN‐γ production by draining lymph node cells from the lesion of BALB/c mice treated with HSP65‐producing *L. lactis* after infection with *L. braziliensis* . BALB/c mice were infected in the ear with *L. braziliensis* metacyclic promastigotes; then, 4 weeks post‐infection, they were fed with water (Lb), GM17 medium containing empty vector‐bearing *L. lactis* (Lb/Ø) or XM17 medium containing HSP65‐producing *L. lactis* (Lb/HSP65) for four consecutive days. From 6 to 10 weeks post‐infection, animals were euthanised, and draining lymph node cells were collected, cultured, and restimulated in vitro with live promastigotes of *L. braziliensis* (1:5). After 48 or 72 h, cell culture supernatants were collected for cytokine quantification by ELISA. (A) IL‐10 production (right panel) and IFN‐γ production kinetics (left panel). (B) The ratio of IL‐10/IFN‐γ production. (A, B) Data are represented by the median and interquartile range for each group. Data are representative of three independent experiments using five animals. *p < 0.05; **p < 0.001.

Cytokine levels were assessed 6 to 10 weeks after *L. braziliensis* infection to evaluate the impact of oral treatment with HSP65‐producing *L. lactis* on the immune profile. Our data showed that animals from the Lb/HSP65 group significantly increased IL‐10 production from 6 to 10 weeks after infection compared to the Lb or Lb/Ø group (Figure 3A). On the other hand, IFN‐γ production was reduced in animals from the Lb/HSP65 group at 6 and 7 weeks after infection, but it increased after 9 to 10 weeks of infection compared to the Lb or Lb/Ø groups (Figure 3B). IL‐4 production was also determined, but no significant differences were observed among groups (data not shown). We measured the non‐active TGF‐β by ELISA, and no differences were observed among the groups; however, the increase of active bound form of TGF‐β could be associated with the increase in regulatory T CD4^+^LAP^+^ cells (Figure 5B).

**FIGURE 4:**
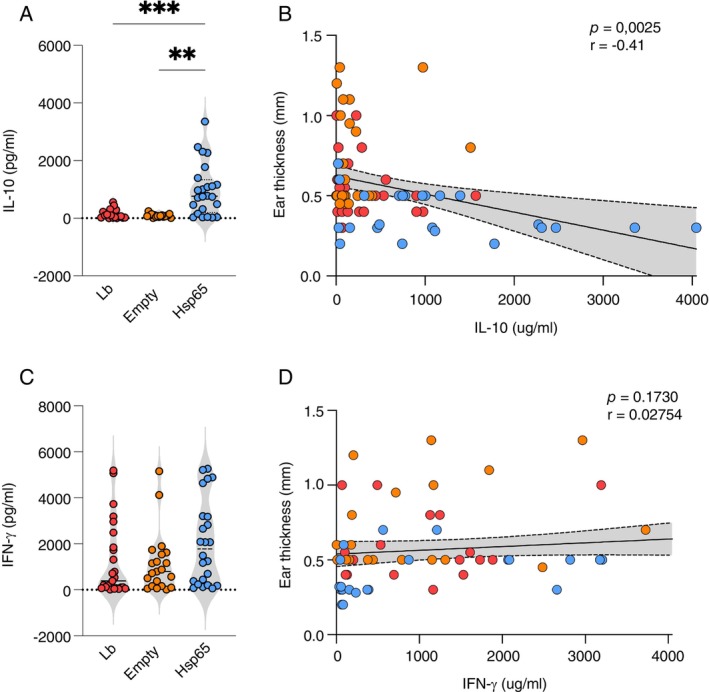
Correlation between IL‐10 production and ear thickness in BALB/c mice treated with HSP65‐producing *L. lactis* after infection with *L. braziliensis* . From 6 to 10 weeks post‐infection, animals were euthanised, and draining lymph node cells were collected, cultured, and restimulated in vitro with live promastigotes of *L. braziliensis* (1:5). After 48 or 72 h, cell culture supernatants were collected for cytokine quantification by ELISA. (A) Pool of IL‐10 production throughout the infection course (all timepoints) of treated or untreated mice. (B) Correlation between IL‐10 production and ear thickness of the lesions from mice infected by *L. braziliensis* and treated or untreated with HSP65‐producing *L. lactis* . (C) Pool of IFN‐γ production throughout the infection course (all timepoints) of treated or untreated mice. (D) Correlation between IFN‐γ production and ear thickness of the lesions from mice infected by *L. braziliensis* and treated or untreated with HSP65‐producing *L. lactis*. Data are represented by the median and interquartile range for each group. The data are representative of three independent experiments using five animals in each group. The Kruskal–Wallis test with Dunn's post‐test was used. *p < 0.05; **p < 0.001.

We also analysed the ratio between IL‐10 and IFN‐γ cytokines to identify the predominant cytokine produced. Mice from Lb/HSP65 showed a higher production of IL‐10 than IFN‐γ during 6 to 8 weeks post‐infection (Figure 3C). Therefore, knowing that IL‐10 is one of the critical cytokines for modulating inflammatory immune responses, we investigated whether the production of IL‐10 and IFN‐γ correlated with the ear thickness. The Lb/HSP65 group produced more IL‐10 than the control groups (Figure 4A). We found a significant negative correlation between high IL‐10 production and smaller ear thickness of lesions in the Lb/HSP65 group (Figure 4B). No correlation between IFN‐γ production and ear thickness was observed (Figure 4C,D). Together, these findings suggest that oral treatment with HSP65‐producing *L. lactis* promoted a modulation between IL‐10 and IFN‐γ production, protecting against tissue damage and control of parasite load.

### 2.4 Oral Administration of HSP65‐Producing *L. lactis* Leads to an Increase in the Treg Cell Frequency in Draining Lymph Nodes of Mice Infected With *L. braziliensis*

**FIGURE 5:**
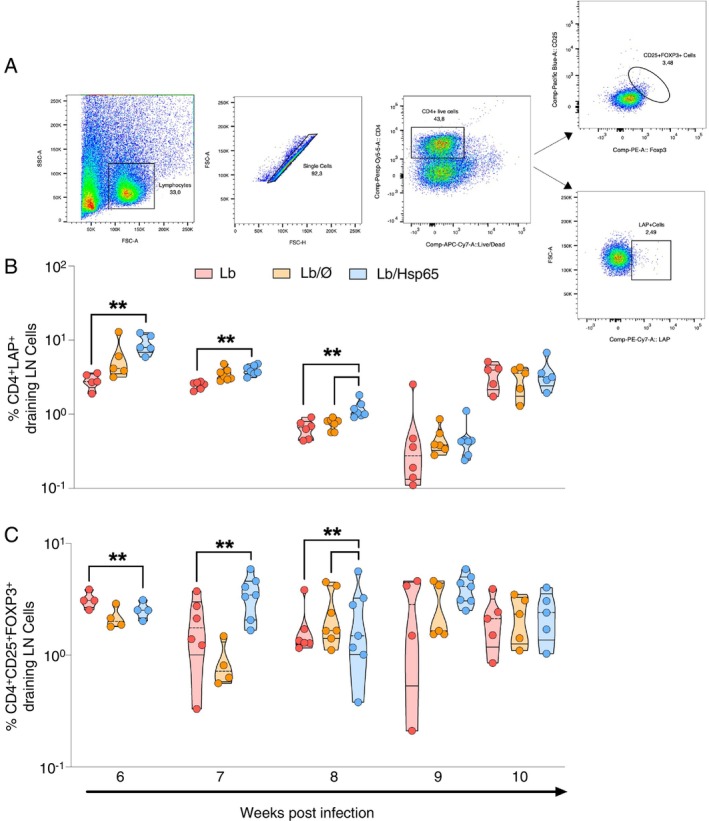
Frequency of CD4^+^LAP^+^ and CD4^+^CD25^+^Foxp3^+^ regulatory T cells in the draining lymph node from the lesion of BALB/c mice treated with HSP65‐producing *L. lactis* after *L. braziliensis* infection. Mice were euthanised at points from 6 to 10 weeks post‐infection, and draining lymph node cells were collected and stained using surface markers (anti‐CD4, anti‐CD25, and anti‐LAP) and an intracellular marker (anti‐Foxp3). (A) Lymphocyte region was delimited by size and granularity (FSC‐A × SSC‐A). Afterwards, the doublet cells were removed by FSC‐A × FSC‐H and viable CD4+ T cells were selected. Within this gate, cells stained with antibodies directed to LAP^+^, CD25^+^ and Foxp3^+^ were determined. (B) Frequency of regulatory T cells CD4^+^LAP^+^ and (C) CD4^+^CD25^+^Foxp3^+^ in the draining lymph node of the lesion at various times post‐infection. Data are represented by the median and interquartile range for each group. The data are representative of three independent experiments using five animals in each group. The Kruskal–Wallis test with Dunn's post‐test was used. *p < 0.05; **p < 0.001.

The frequencies of T CD4^+^CD25^+^Foxp3^+^ and CD4^+^LAP^+^ cells were investigated by flow cytometry in draining lymph nodes weekly during the infection. At 6‐, 7‐, and 8‐weeks post‐infection, animals from the Lb/HSP65 group showed a higher frequency of CD4^+^LAP^+^ T cells when compared to their control groups (Figure 5A). Likewise, a higher CD4^+^CD25^+^Foxp3^+^ T cell frequency was observed in animals from the Lb/HSP65 group only at 7 weeks post‐infection (Figure 5B). From 9 to 10 weeks post‐infection, there were no significant differences in the frequencies of CD4^+^LAP^+^ T cells and CD4^+^CD25^+^Foxp3^+^ T cells among the experimental groups (Figure 5A,B).

### 2.5 Oral Administration of HSP65‐Producing *L. lactis* Did Not Mitigate Inflammation in BALB/c Mice Infected With *L. major*

**FIGURE 6:**
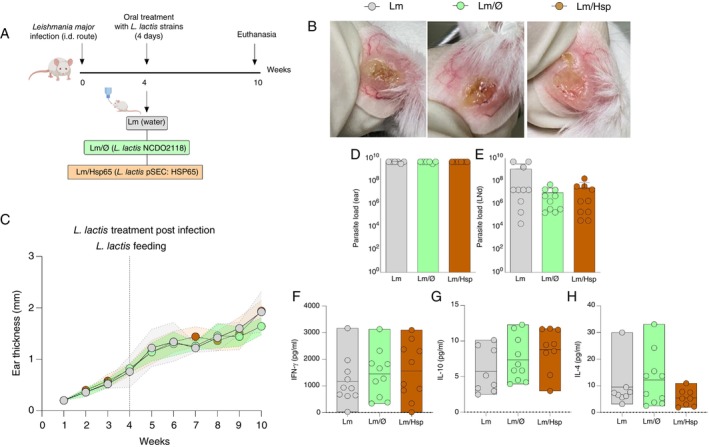
Oral administration of HSP65‐producing *L. lactis* did not mitigate inflammation in BALB/c mice infected with *L. major* . BALB/c mice were challenged in the left ear with 5 × 10^5^ metacyclic promastigotes of *L. major* via intradermal injection. Four weeks post‐infection, the animals were orally administered with water (Lm), GM17 medium containing empty vector‐bearing *L. lactis* (Lm/Ø) or XM17 medium containing HSP65‐producing *L. lactis* (Lm/HSP65) for four consecutive days. Ear thickness was measured weekly with an analogue calliper. (A) Experimental design in BALB/c mice. (B) Lesion development caused by *L. major* infection in BALB/c mice from the Lm, Lm/Ø, and Lm/HSP groups. Lesions were photographed at 7 weeks post‐infection for macroscopic analysis. (C) Kinetics of lesion thickness in the Lm, Lm/Ø and Lm/HSP groups. Data represent the means ± standard deviation (SD) of each group, and a one‐way ANOVA test was used for comparison among multiple groups. (D, E) Kinetics of parasite load in the ear and draining lymph node of BALB/c mice in the Lm, Lm/Ø and Lm/HSP groups. Ten weeks post‐infection, the animals were euthanised, and the ears and draining lymph nodes were collected for parasite quantification by limiting dilution. Kinetics of parasite load in the ear (D) and the draining lymph node (E). Data are represented by the median and interquartile range for each group. The Kruskal–Wallis test with Dunn's post‐test was used for multiple comparisons. (F, G) Kinetics of pro‐inflammatory and anti‐inflammatory cytokine production (IL‐10 and IFN‐γ) from draining lymph node cells in BALB/c mice of the Lm, Lm/Ø and Lm/HSP groups. Cells obtained from the draining lymph nodes were cultured and re‐stimulated in vitro with metacyclic promastigotes of *L. major* . Supernatants were collected and stored for cytokine quantification by ELISA. Kinetics of IL‐10 (F) and IFN‐γ (G) production. Data are represented by the median and interquartile range of each group. The Kruskal–Wallis test with Dunn's post hoc test was used.

To evaluate the potential immunomodulatory effects of orally administered *L. lactis* producing HSP65 in a severe *Leishmania* infection model, we infected BALB/c mice with *L. major* , a strain known for causing progressive severe lesions in this mouse lineage [4]. Four weeks after infection, mice were divided into groups and fed with water (Lm), GM17 medium containing *L. lactis* with an empty vector (Lm/Ø), or XM17 medium containing HSP65‐producing *L. lactis* (Lm/HSP65) for four consecutive days, from day 28 to 31 post infection (Figure 6A). All groups developed severe lesions characterised by intense inflammation, ulcer formation and necrosis (Figure 6B). Interestingly, no significant differences in ear thickness were observed, indicating that HSP65‐producing *L. lactis* treatment did not mitigate inflammation caused by *L. major* (Figure 6C).

We further explored the effect of HSP65‐producing *L. lactis* on parasite load from ears and lymph nodes on parasitic immunity in these mice. The results showed no significant reduction in parasite load in the ears and draining lymph nodes among the groups (Figure 6D,E). Regarding IFN‐γ, IL‐10 and IL‐4 production, there was no statistically significant difference between the Lm/HSP65 group and the others (Figure 6F–H).

## 3 Discussion

Herein, we examined the effect of HSP65‐producing *L. lactis* in mice already infected with *L. braziliensis* to investigate whether the modulatory mechanisms induced by oral tolerance would be effective in infected mice. Draining lymph node cells from *L. braziliensis* ‐infected and HSP65‐treated mice produced more IL‐10 than IFN‐γ and developed smaller lesions than the untreated mice. In contrast, Lb and Lb/∅ groups exhibited intense tissue destruction and ulceration, indicating an exacerbated cellular immune response characteristic of the CL model [27, 28, 29]. However, when we analysed its effect in a more severe and progressive infection model using *L. major* ‐infected BALB/c mice, oral administration of HSP65‐producing *L. lactis* did not affect lesion development nor parasite load, presenting progressive and severe lesions in all animals. This suggests that oral treatment with HSP65‐producing *L. lactis* only affects *L. braziliensis* infection, since HSP65‐treated animals produced enough IFN‐γ, able to stimulate the leishmanicidal effect of the macrophages [3, 30, 31], thus reducing the parasite load. The OT possibly creates an anti‐inflammatory environment with the increase of IL‐10 and rise in Tregs frequency, that could affect the inflammation as shown in other non‐pathogenic inflammatory diseases [23, 24, 25]. This nuanced cytokine balance appears sufficient to control parasite replication without causing extensive tissue damage, as observed in subclinical individuals [32]. Similar findings in the atherosclerosis model support our data, which showed that HSP65‐induced oral tolerance led to decreased disease severity through increased IL‐10 and reduced IFN‐γ production [20].

In contrast, *L. major* infected BALB/c mice, treated with HSP65‐ *L. lactis* showed no significant differences in IL‐10 and IFN‐γ production, as well as IL‐4 production, suggesting that the inflammatory response in CL caused by *L. major* is driven by distinct underlying mechanisms such as early higher production of IL‐4 and IL‐10 and lower production of IFN‐γ [33, 34], allowing parasite proliferation.

In the CL model by *L. braziliensis* , we observed an anti‐inflammatory effect, potentially caused by the significant increase in CD4^+^LAP^+^ and CD4^+^CD25^+^Foxp3^+^ T cell frequencies in draining lymph nodes from 6 to 8 weeks post‐infection in mice from Lb/HSP65 compared to Lb and Lb/Ø groups. These regulatory T cell subsets play a crucial role in inducing an immunomodulatory profile that reduces inflammation in ear lesions. Continuous oral administration of antigens has been reported to induce regulatory T cells that can mitigate inflammation in models of autoimmune diseases [10]. Generating regulatory T cells as well as anti‐inflammatory cytokines is crucial for oral tolerance induction [6]. These cells, particularly CD4^+^LAP^+^ T cells expressing membrane‐bound TGF‐β, may migrate from the intestinal mucosa to peripheral lymphoid organs, contributing to immune regulation, as observed in experimental autoimmune encephalomyelitis [23]. Our study revealed an increased frequency of CD4^+^LAP^+^ T cells in mice from the Lb/HSP65 group. However, no significant changes in overall TGF‐β production were noted (data not shown), suggesting that the active form of TGF‐β may be membrane‐bound in these cells within draining lymph nodes. Prior studies have demonstrated that HSP65‐producing *L. lactis* prevents arthritis [25] and colitis [24] in mice dependent on mechanisms such as TLR2 and induction of these regulatory T cells. We also observed reduced inflammation and an increased frequency of these cells in mice treated with HSP65 prior to *L. braziliensis* infection. This immunomodulatory effect was dependent on TRL2, LAP and IL‐10 [26], reinforcing the role of these Tregs induced by oral tolerance in suppressing inflammatory responses [10].

We observed the generation and effectiveness of regulatory mechanisms induced by oral administration of HSP65‐producing *L. lactis* after priming with the infectious agent. This is remarkable for two reasons. First, it has been described that oral tolerance mechanisms are very effective as a preventive modulatory tool but are less effective in sensitised animals [35]. It is likely that *L. braziliensis* infection, a self‐healing infection, sets the stage for the induction of regulatory mechanisms that are generated by the oral administration of HSP65‐producing *L. lactis*. In addition, a therapeutic effect of HSP65‐producing *L. lactis* indicates that this recombinant probiotic may have application in a clinical setting in which individuals and animals are already infected. In contrast, *L. major* induces a severe infection in BALB/c mice, a non self‐healing infection, which could not be modulated by HSP65‐producing *L. lactis* oral treatment since the infection induces a strong Th2‐dominated response, characterised by early elevated production of IL‐4 and IL‐10, which contributes to increased susceptibility and progressive ulceration in BALB/c mice [4, 34, 36, 37, 38, 39, 40]. The lesion dynamics further illustrate these differences; mice infected with *L. braziliensis* develop small, self‐healing nodular lesions [27], whereas those infected with *L. major* present with progressive, non‐healing ulcers [39]. It is plausible that the administration of HSP65‐producing *L. lactis* anticipates these regulatory mechanisms (IL‐10 production and Treg generation) in *L. braziliensis* infection but cannot do the same in *L. major* infection. The number of parasites is high in lesions from BALB/c mice infected by *L. major* ; therefore, IFN‐γ is necessary to activate the macrophages to kill the intracellular parasites. However, it would be interesting to test different protocols, using different time points after the infection and for a more prolonged time of treatment. In the early infection by *L. braziliensis* (6 and 7 weeks), the Lb/HSP65 group exhibited acute inflammation that could efficiently resolve the infection, and a decreased inflammation intensity in the later infection (8 to 10 weeks) may protect mice from chronic tissue damage, although no significant differences were observed in histopathological scores.

Resistance to infection is a function of the immune system, which acts by detecting, neutralising, and destroying pathogens, reducing the pathogen burden. Both innate and adaptive immune systems contribute to infection resistance [41]. A favourable immune response is a result of the balance between an acceptable level of immunopathology and pathogen elimination [42]. Indeed, the factor required to reduce the burden of the pathogen is the same that causes immunopathology [43]. Tolerance to the pathogen reduces the negative impact of an infection on the host without directly affecting parasite load [44, 45, 46].

Heat shock proteins (HSPs) have demonstrated significant potential in tissue preservation across multiple autoimmune disease models [11, 21, 22, 23]. Our findings support this concept by showing that a balanced immune response can effectively eliminate pathogens while minimising host damage [44]. However, while our results demonstrate the efficacy of recombinant *L. lactis* treatment in *L. braziliensis* infection, this approach showed limited effectiveness against *L. major* , highlighting important species‐specific limitations. Our work highlights the therapeutic potential of oral tolerance induction using HSP65‐producing *L. lactis* , particularly for parasite infections in which immune responses exhibit a primed balance. This strategy may offer a promising alternative for targeted treatment of specific *Leishmania* infections.

## 4 Material and Methods

### 4.1 Animals

Female BALB/c mice (6–8 weeks old) were used under ethical approval (CEUA no. 003/2019 and 032/2023, IGM/Fiocruz‐Bahia). Mice were housed in a pathogen‐free facility at IGM‐FIOCRUZ, Brazil.

### 4.2 Parasites

*L. braziliensis* (MHOM/BR/01/BA788) and *L. major* (MRHO/SU/59/P/LV39) promastigotes were cultured in Schneider's medium (Sigma) with 20% FBS (fetal bovine serum), L‐glutamine (2 mM), penicillin (100 U/mL) and streptomycin (100 μg/mL) at 24°C for 5 days until the stationary phase.

### 4.3 Experimental Infection and Mice Follow Up

Metacyclic *L. braziliensis* promastigotes were purified using the lectin *Bauhinia purpurea* lectin [47]; *L. major* was purified via Ficoll gradient [48]. Mice were infected intradermally in the left ear with 5 × 10^5^ metacyclic promastigotes in 10 μL of saline. Lesion progression was monitored weekly using a digital calliper (Thomas Scientific, USA).

### 4.4 Generation of HSP65‐Producing *Lactococcus lactis*

A recombinant *L. lactis* strain NCDO2118 (provided by Dr. Faria and Dr. Azevedo) secreting *M. leprae* HSP65 (pSEC: HSP65) was generated using a xylose‐inducible system (XIES) [49]. Control strains included empty‐vector *L. lactis* (pXylT:SEC).

### 4.5 *L. lactis* Strains Growth and Xylose Induction

*L. lactis* was grown in M17 broth (Difco) with 0.5% glucose (GM17) or 1% xylose (XM17) at 30°C. For HSP65 induction, cultures were diluted 1:10000 in XM17 + chloramphenicol (10 μg/mL). After 3 days, cultures achieved an optical density of 2.0 at a wavelength of 600 nm, corresponding to 2.5 × 10^8^ CFU/mL. Cultures were stored in 80% glycerol at −80°C.

### 4.6 Oral Administration of *L. lactis* Strains

BALB/c mice at 4 weeks of infection caused by *L. braziliensis* or *L. major* were randomly distributed into three experimental groups: mice fed with water (Lb), GM17 media with empty‐vector‐bearing *L. lactis* (Lb/Ø), and XM17 media containing *M. leprae* HSP65‐producing *L. lactis* (Lb/HSP65). Each mouse received 5 mL of *L. lactis* culture (GM17 or XM17) per day at a concentration of 2.5 × 10^8^ CFU/mL during 4 consecutive days, from day 28 to 31 post infection [23, 25, 26, 50]. We initiated the treatment at this point because the lesions had already developed. We performed five experiments, using three different groups of animals as described above, and each group was composed of five animals. For *L. major* experiments, we performed three experiments, with 3 different groups and 10 mice per group.

### 4.7 Parasite Load Quantification

At 6–10 weeks post‐infection, ears and lymph nodes were collected, macerated individually, and subsequently centrifuged at 1962 *g*/10 min. Parasite burden was assessed via limiting dilution assay, previously described by Belkaid et al. [37, 51, 52].

### 4.8 Cell Cultures and Cytokines Production Assay

Draining lymph node cells from the lesions (1 × 10^6^/well) were cultured at 37°C under 5% CO_2_ with *L. braziliensis* or *L. major* at a proportion of a cell‐to‐parasite ratio of 1:5 and ConA (5 μg/mL) as a positive control. After 48 or 72 h, supernatants were collected and IL‐10, IFN‐γ, IL‐4 and TGF‐β were measured by ELISA (BD Biosciences).

### 4.9 Flow Cytometry for T Regs Characterisation

Cells suspensions from draining lymph nodes were obtained, washed with FACS Buffer (PBS + 1% SBF) at 1800 rpm for 5 min at 4°C and stained with anti‐CD4 (Percp‐Cy5.5, Clone RM4‐5, eBioscience), anti‐CD25 (eFluor450, Clone 3C7, eBioscience), anti‐LAP (PE‐Cy7, Clone TW7‐16B4, eBioscience) and fixable viability dye (eFluor780, eBioscience). Subsequently, cells were washed and pre‐incubated with a fixation/permeabilisation solution (BD Biosciences, USA). For intracellular staining, cells were labelled with anti‐Foxp3 (PE, Clone FJK16s, eBioscience). Flow cytometry analysis was performed on a FACS Fortessa (FACS, BD Biosciences) using FlowJo software, version 10.5.0 (Tree Star Inc.). To perform the flow analysis, 100.000 events were acquired per sample. Initially, the lymphocyte region was delimited by size and granularity (FSC‐A × SSC‐A). Afterwards, the doublet cells were removed by FSC‐A × FSC‐H and viable CD4^+^ T cells were selected. Within this gate, the cells population stained with antibodies directed to LAP^+^, CD25^+^, Foxp3^+^ were determined (Figure 5A).

### 4.10 Histopathology Analysis

Mouse ears from all experimental groups were removed 6–10 weeks post infection. Ears were fixed in 10% paraformaldehyde, processed, sectioned (5 μm), and stained with Haematoxylin and Eosin (H&E). Inflammation intensity and the presence of inflammatory cell infiltrates were scored from 0 to 3, according to each parameter based on their intensity, with a score of 0 indicating absence, 1 indicating presence of 1%–25%, 2 indicating the presence of 25%–50%, and 3 indicating presence of > 50%. The scores for each animal's evaluated parameters were corrected based on their respective scores for inflammation intensity.

### 4.11 Statistical Analysis

Data normality was assessed via D'Agostino‐Pearson. Parametric data (ANOVA + Tukey's) and non‐parametric data (Kruskal–Wallis + Dunn's) were analysed. To evaluate disease burden in each mouse, ear thickness and parasite loads were recorded weekly in all treated and control mice. These parameters were plotted individually for experimental and control mice throughout the course of infection. Disease burden was calculated as the Area Under the Curves (AUC) in plots of ear thickness and parasite load. Results are presented as means ± standard deviation (SD) or medians and interquartile range (IQ). *p* values < 0.05 were assigned as statistically significant. All statistical analyses were performed using GraphPad Prism v.9 software (San Diego, CA, USA).

## Conflicts of Interest

The authors declare no conflicts of interest.

## Supporting information


**Figure S1:** imm70022‐sup‐0001‐Figures.docx.
**Figure S2:** imm70022‐sup‐0001‐Figures.docx.

## Data Availability

The data that support the findings of this study are available from the corresponding author upon reasonable request.
