# Supplementary material for: Oral Treatment With Heat Shock Protein 65‐Producing Lactococcus lactis Induces Regulatory T Cells, Modulating Inflammatory Response in Leishmania braziliensis Infection
Source: Immunology. 2025 Jul 31;177(1):59–69. doi: 10.1111/imm.70022 (PMC12665804; doi:10.1111/imm.70022)
Supplement: Supplementary file 1 — Figure S1: imm70022‐sup‐0001‐Figures.docx. Figure S2: imm70022‐sup‐0001‐Figures.docx. [file IMM-177-59-s001.docx]

**SUPPLEMENTARY MATERIAL**


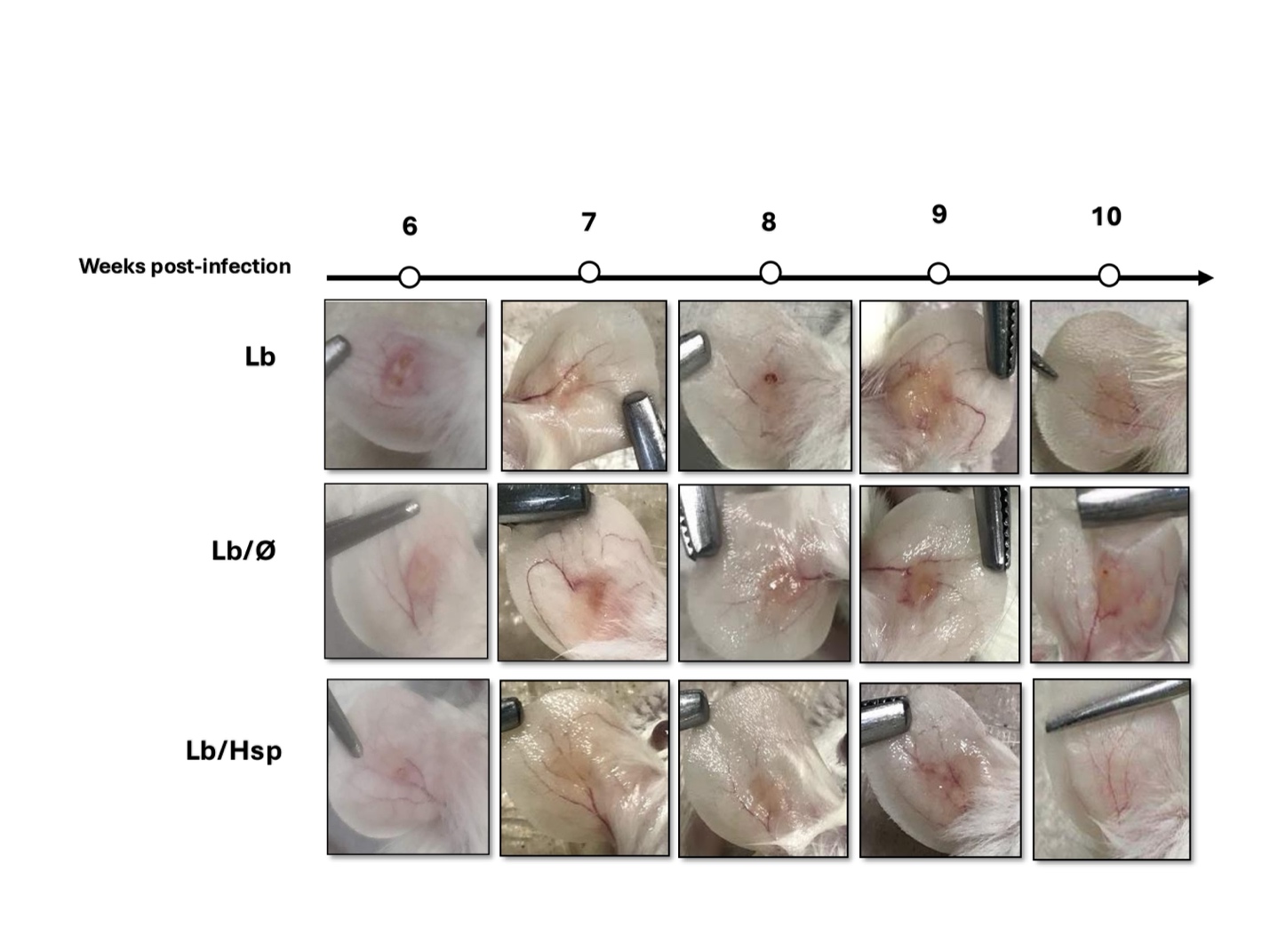


**Supplementary Figure 1. Macroscopic analysis from ear lesions of mice treated or not with HSP65-producing *L. lactis* after *L. braziliensis* infection.** BALB/c mice were infected in the ear with *L. braziliensis* metacyclic promastigotes then, four weeks post-infection, fed with water (Lb), GM17 medium containing empty vector-bearing *L. lactis* (Lb/Ø) or XM17 medium containing HSP65-producing *L. lactis* (Lb/HSP65) for four consecutive days. (A) Representative lesion macroscopic aspects from 6 to 10 weeks after infection in respective animal groups.


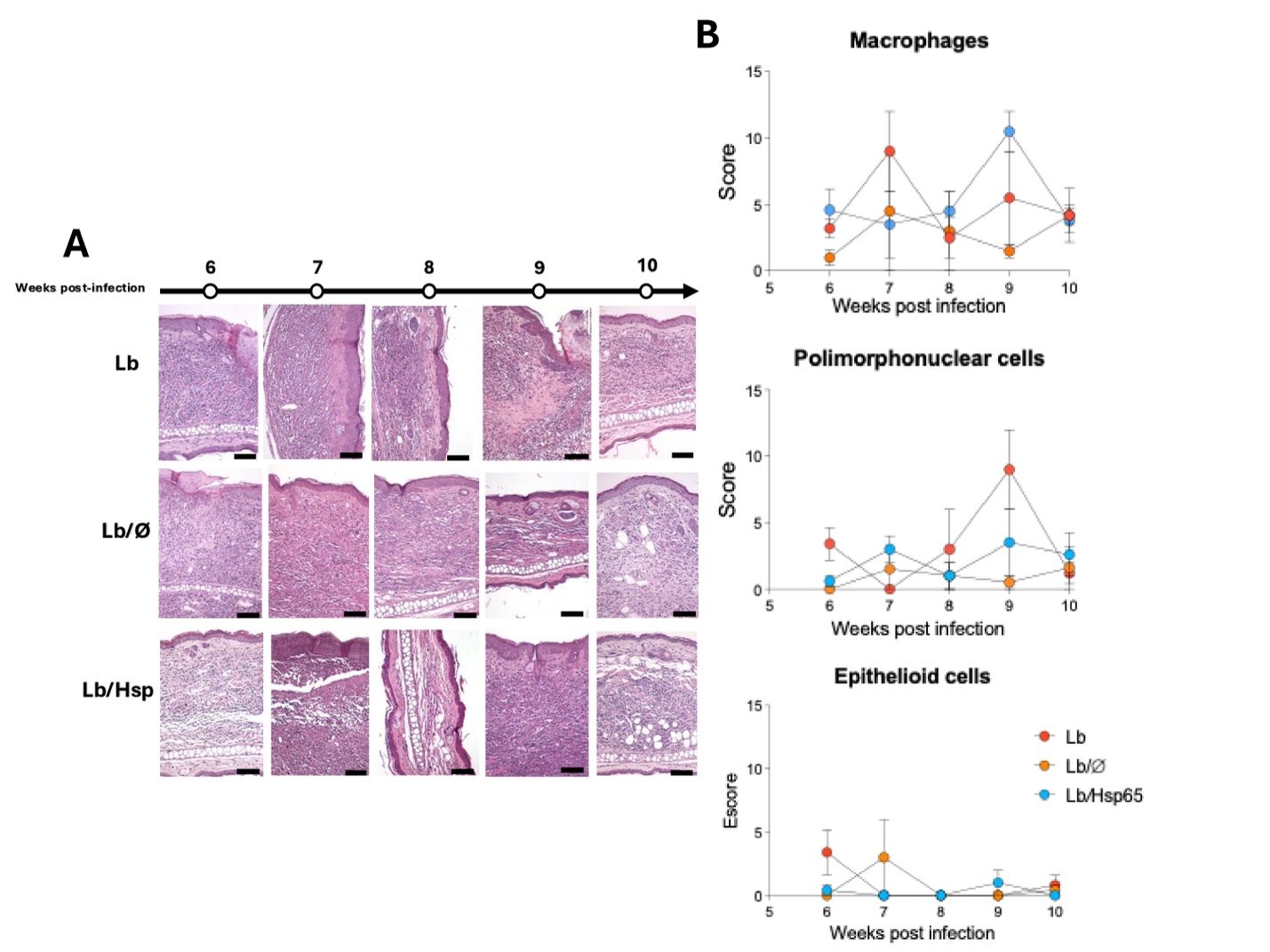


**Supplementary Figure 2. Histopathological score analysis from ear lesions of mice treated or not with HSP65-producing *L. lactis* after *L. braziliensis* infection.** BALB/c mice were infected in the ear with *L. braziliensis* metacyclic promastigotes then, four weeks post-infection, fed with water (Lb), GM17 medium containing empty vector-bearing *L. lactis* (Lb/Ø) or XM17 medium containing HSP65-producing *L. lactis* (Lb/HSP65) for four consecutive days. (A) Representative lesion histopathological from 6 to 10 weeks after infection in respective animal groups. Ears were collected, fixed in 10% formaldehyde, processed, then stained with hematoxylin and eosin. (B) Macrophages, polymorphonuclear and epithelioid cells (score kinetics. Histopathological changes were evaluated through score kinetics. We determined the score according to intensity: 0 (absence), 1 (presence of 1-25%), 2 (presence of 25-50%), and 3 (> 50%). We used a healthy animal ear as a negative control. Data are represented by the median and interquartile range for each group. The data are representative of three independent experiments.
